# Chia-Chen Tan and genetics in modern China

**DOI:** 10.1007/s13238-018-0539-2

**Published:** 2018-04-25

**Authors:** Lei Fu

**Affiliations:** Zhejiang Normal University, Jinhua, 321004 China

**Figure 1:**
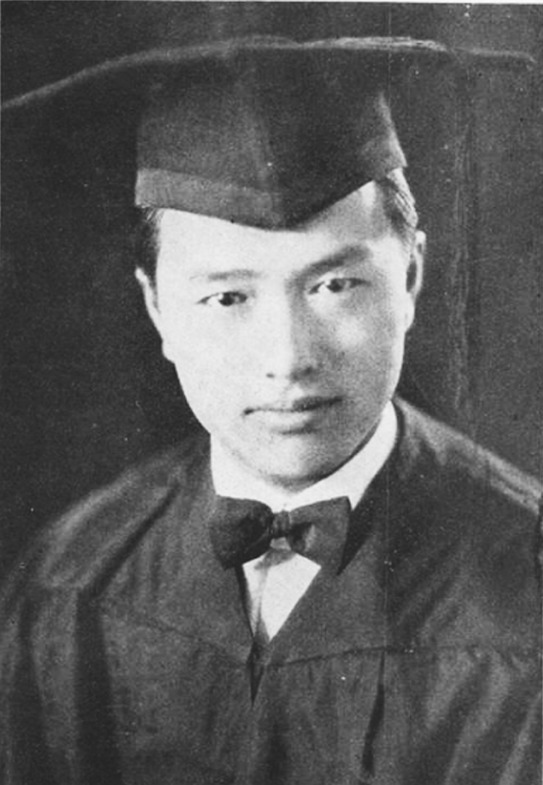
Chia-Chen Tan (1909–2008)

Chia-Chen Tan (谈家桢, 1909–2008) was one of the most important founders of genetics in modern China and made great effort to the internationalization of Chinese genetics (Fig. 1).

**Figure 2:**
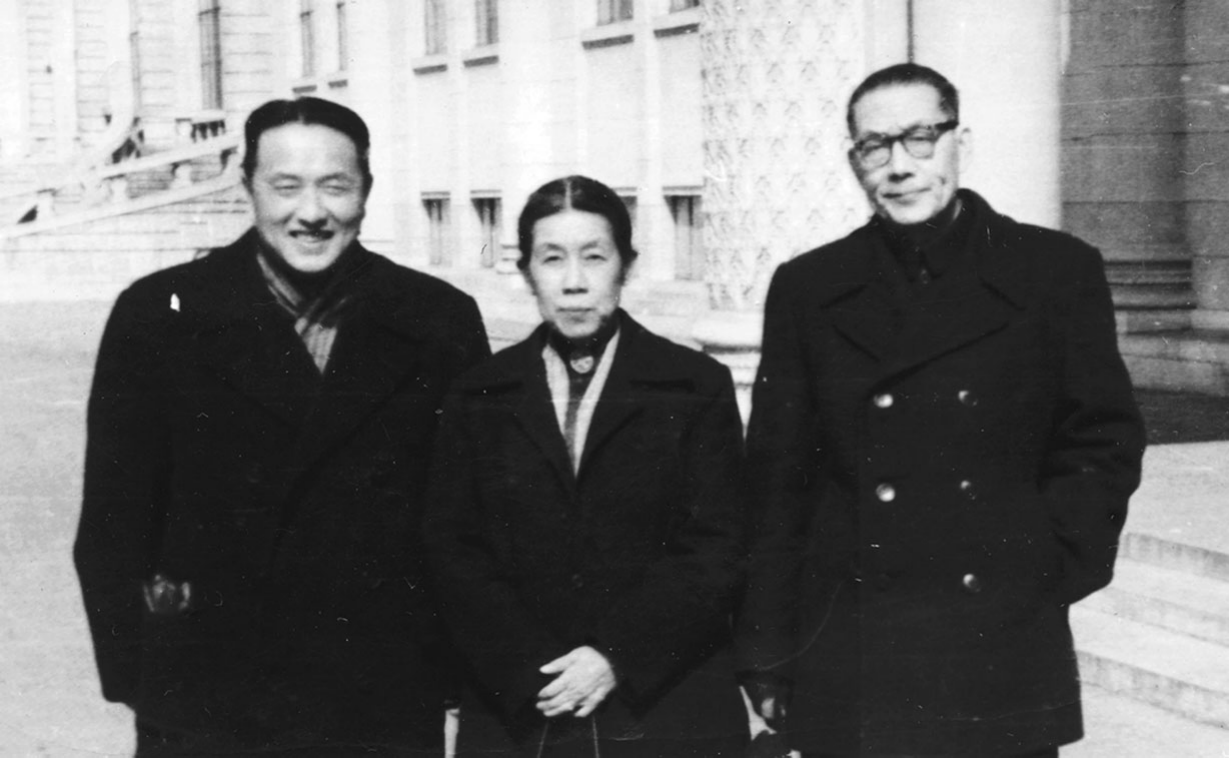
Chia-Chen Tan (Left) and Mr. and Mrs. Ju-Chi Li

Chia-Chen Tan was born on September 15, 1909 in Zhejiang Province. He studied in missionary middle school and public high school successively before being admitted to Soochow University without examination. He majored in biology in university and became interested in genetics. After graduating in 1930, he entered YenChing University and learnt from Ju-Chi Li (李汝祺) (Fig. 2) who was the first Chinese student to receive a doctor’s degree from American famous geneticist Thomas Hunt Morgan and was the only genetics professor in YenChing University at that time (Zhang, 2017). Following Wu Chenfu Francis’s (胡经甫) suggestion and Ju-Chi Li’s instruction, Chia-Chen Tan completed his master research on lady-bird genetics with several excellent articles. Ju-Chi Li recommended his article to Thomas Hunt Morgan whose lab was famous for research on inheritance and variation in fruit flies. His outstanding work got Morgan’s attention and appreciation, which promoted their cooperation later. After working at Soochow University for several months, Tan went abroad to Morgan’s lab in 1934 for further study and he got Ph. D. from California Institute of Technology in 1936 under the supervision of Morgan and Dobzhansky. In the following years, he published dozens of articles in international journals, which made him known by the world. Then he declined Morgan’s detainment and returned to China, being appointed professor in Chekiang University. During Anti-Japanese War, Tan continued his research on lady-bird and cultivated his first graduate students in Meitan, Guizhou Province. He then became head of biology department in 1952 and director of institute of genetics in 1961 in Fudan University. He was elected academician of the Chinese Academy of Sciences in 1980.

As an outstanding and prolific scientist, Chia-Chen Tan worked on several fields of genetics and made a large number of world-famous findings. When he was in YenChing University, Chia-Chen Tan worked on inheritance and variation in the color of patterns in the lady-bird beetles. Even during war times, he continued his researches on genetics of lady-bird and fruit flies. It was in this period that he made world-famous discovery about mosaic dominant inheritance of color patterns in the lady-bird beetles, which is still a classical example in modern genetics textbooks. In 1940s, he went to USA again to continue his research on mosaic dominance in the inheritance of color patterns in the lady-bird beetles, which had a significant impact on international genetics (Tan, 1946). He considered lady-bird beetles as good model of micro-evolution. Tan figured out the genetic maps of autosomes in *Drosophila pseudoobscura* when he was in Morgan’s lab. Then he proved how gene evolved through repetition and differentiation by investigating the nature of the race-differential chromosomes in *Drosophila montium* (Tan, 1942). From 1960s he paid attention to radiation cytogenetics. His team chose *Macaca mulatta* as experimental material and tested the effect of X-ray and γ-ray to *Macaca mulatta*. They expanded their research to environmental toxicology which was pioneering in China. Later in his life, Tan suggested the government supporting researches on human genome for the sake of protection of Chinese human gene resources. Thanks to his advice, China established two national genome research centers. In his 70 years of teaching and researching, Tan published more than 100 articles, which are valuable treasure for science. Tan served as president of the Genetics Society of China, Chinese Environmental Mutagen Society and Chinese Society of Biotechnology and made substantial contributions to the development of the related disciplines.

Chia-Chen Tan participated actively in international cooperation. He was the first to introduce the term “gene” to China. As early as 1945–1946, he went to USA again as visiting professor. In 1948 he was elected member of council of the 8th International Congress of Genetics. After a few decades, he visited America in 1978 and introduced the progress of genetics in China. And then he invited several American professors to China to lecture on molecular genetics. From 1980s to 1990s more and more scientists from America and Europe came to China for academic communication at his invitation. He was the leading proponent and chairman of the 18th International Congress of Genetics held in Beijing in 1998. Besides, more and more Chinese scholars went abroad for further research under his aid. He was awarded outstanding alumni by California Institute of Technology, Honorary Doctoral Degree by York University, Canada and Honorary Fellow of Japanese Society of Genetics. He was member of National Academy of Sciences of USA, Lincean Academy of Italy and The Third World Academy of Sciences (Zhao,1998). Under efforts of Chia-Chen Tan and other excellent scientists, Chinese genetics and other sciences became known to the world.

Chia-Chen Tan devoted himself to science education and cultivated several distinguished graduate students including Shi Lvji (施履吉), Sheng Zujia (盛祖嘉), Liu Zudong (刘祖洞), Xu Daojue (徐道觉) and so on. He considered the basics of knowledge, theories and experimental skills important for students. He supported his students to go abroad for further studies. More than that, He invited most of them to come back to work in China. He was continuously keen on science popularization and wrote several books and articles for public, such as *Secret of Life*, *Gene and Heredity*, *Mendel and His Science Career*, *A Biography for Biologists in Modern China* and so on, in which he laid stress on history of science and technology.

Chia-Chen Tan is a world-renowned scientist. In 1999 the No. 3542 asteroid discovered by Purple Mountain Observatory, Chinese Academy of Sciences in 1964 was named 3542 Tanjiazhen, which is in honor of Chia-Chen Tan’s contribution to science.
